# Link of sorafenib resistance with the tumor microenvironment in hepatocellular carcinoma: Mechanistic insights

**DOI:** 10.3389/fphar.2022.991052

**Published:** 2022-08-22

**Authors:** Xinchen Tian, Tinghao Yan, Fen Liu, Qingbin Liu, Jing Zhao, Huabao Xiong, Shulong Jiang

**Affiliations:** ^1^ Cheeloo College of Medicine, Shandong University, Jinan, China; ^2^ Clinical Medical Laboratory Center, Jining First People’s Hospital, Jining Medical University, Jining, China; ^3^ Institute of Immunology and Molecular Medicine, Basic Medical School, Jining Medical University, Jining, China

**Keywords:** tumor microenvironment, hepatocellular carcinoma, sorafenib resistance, tumor-associated immune-suppressive cells, immunosuppressive factors, hypoxia

## Abstract

Sorafenib, a multi-kinase inhibitor with antiangiogenic, antiproliferative, and proapoptotic properties, is the first-line treatment for patients with late-stage hepatocellular carcinoma (HCC). However, the therapeutic effect remains limited due to sorafenib resistance. Only about 30% of HCC patients respond well to the treatment, and the resistance almost inevitably happens within 6 months. Thus, it is critical to elucidate the underlying mechanisms and identify effective approaches to improve the therapeutic outcome. According to recent studies, tumor microenvironment (TME) and immune escape play critical roles in tumor occurrence, metastasis and anti-cancer drug resistance. The relevant mechanisms were focusing on hypoxia, tumor-associated immune-suppressive cells, and immunosuppressive molecules. In this review, we focus on sorafenib resistance and its relationship with liver cancer immune microenvironment, highlighting the importance of breaking sorafenib resistance in HCC.

## 1 Introduction

According to the latest global cancer statistics, primary liver cancer (PLC) is the sixth most common diagnosed cancer and the third leading cause of cancer death worldwide, with report of 830,000 deaths in 2020 (Sung et al., 2021). Hepatocellular carcinoma (HCC) is the most common histologic type of PLC, accounting for approximately 80–90% (Yang W et al., 2019; Zhu W et al., 2020). Established risk factors for HCC include hepatitis B and C virus infections, alcohol consumption, smoking, obesity, and aflatoxin contamination (Ma et al., 2019). Currently, multidisciplinary treatment strategies are used for HCC, including surgical resection, liver transplantation, transcatheter arterial chemoembolization (TACE), chemotherapy, radiotherapy and molecular targeted therapy (Gao Y et al., 2019). However, HCC is a highly invasive and metastatic tumor with insidious early clinical manifestations. Therefore, most HCC patients lose the opporet tunity for surgery or acquire poor surgical outcomes (Dang et al., 2017). Systemic therapy becomes only treatment option for these patients. Meanwhile, sorafenib has been recognized as a standard first-line treatment for advanced HCC (Pinyol et al., 2019).

Sorafenib, an oral multi-kinase inhibitor, was permitted by the U.S. Food and Drug Administration (FDA) for the treatment of renal cell carcinoma, HCC, and thyroid cancer in 2009 (Yang and Stockwell, 2016). Sorafenib exerts anti-proliferation and anti-angiogenesis effects by inhibiting various kinases, such as inhibiting the tyrosine kinase activity of the cell surface: vascular endothelial growth factor receptor (VEGFR) family, platelet-derived growth factor receptor (PDGFR) family, hepatocyte factor receptor (c-Kit) and FMS-like tyrosine kinase (FLT-3) and suppressing the intracellular Raf family kinase (Yang and Stockwell, 2016). According to the phase III sorafenib Asia-Pacific (AP) trial and the sorafenib HCC Assessment Randomized Protocol (SHARP) trial, sorafenib was effective in prolonging 3 months of median overall survival in patients with late-stage HCC (Llovet et al., 2008; Cheng et al., 2009). However, the therapeutic effect of sorafenib is mainly limited by drug resistance. Only about 30% of HCC patients acquired benefits from sorafenib and the resistance always arose within 6 months in HCC patients (Ford et al., 2009). Hence, it is critical to identify underlying mechanisms and effective strategies to improve its therapeutic outcome.

Tumorigenesis is a complex process requiring synergistic changes in both tumor cells and tumor microenvironment (TME) (Franco et al., 2015). TME has been shown to be critical for tumor progression (Yao et al., 2020) and the development of drug resistance (Borden, 2014). TME was usually classified into cellular and non-cellular components, both of which have been reported to significantly influence drug resistance. In this review, we discuss the relationship between different immune-associated components of TME and sorafenib resistance and provide potential targets that could improve the resistance.

## 2 Sorafenib resistance

**FIGURE 1:**
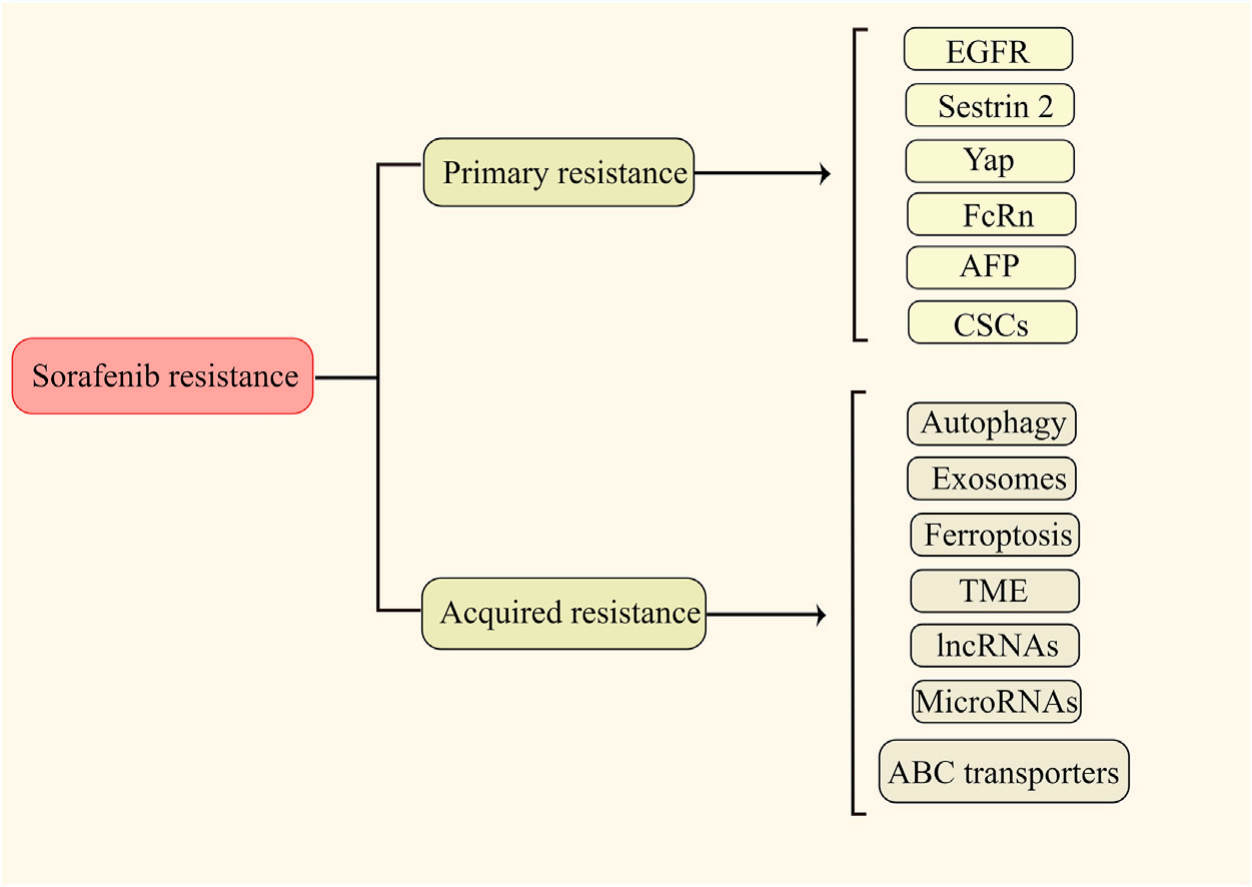
Major determinants of the primary and acquired resistance (edited by Figdraw software).

It has widely been accepted that sorafenib resistance is classified as primary (intrinsic) and secondary (acquired) resistance. Primary resistance denotes that due to the genetic heterogeneity of tumor cells, liver cancer cells already have the resistance factor(s) before sorafenib treatment, which leads to the insensitivity of sorafenib at the early stage of treatment (Xu et al., 2019). Acquired resistance refers to the phenomenon that tumor cells become less sensitive to sorafenib after a period of treatment, resulting in treatment failure (Niu et al., 2017; Yu et al., 2020). Because primary and secondary resistances greatly limit the therapeutic effect of sorafenib, it is important and necessary to gain an in-depth understanding of them. Published resistance mechanisms are presented in Figure 1.

### 2.1 Primary resistance

The molecular mechanisms behind the primary resistance of sorafenib are poorly understood. Based on previous reports, the primary resistance are mainly associated with following molecules and cellular change: 1) EGFR (epidermal growth factor receptor) belongs to the ErbB/EGFR family of receptor tyrosine kinases (RTKs) (Yang Y. M. et al., 2020). The binding of EGFRs to their ligands triggers a series of downstream signaling pathways to promote cell proliferation, survival, and invasion (Tan et al., 2020). Ezzoukhry et al. found that activating EGFR contributed to the primary resistance of sorafenib in HCC by activating the RAF-MEK-ERK cascade. Either inhibiting the kinase activity of EGFR or down-regulating its expression significantly increased the effects of sorafenib on the resistance cells (Ezzoukhry et al., 2012). 2) Sestrin 2 is a probable biomarker and therapeutic target for tumor treatment that plays a vital role in the incidence and development of malignance. Some studies identified sestrin 2 as a tumor suppressor gene, while others described it as an oncogene (Qu et al., 2021). A previous study found that the expression of SESN2 was positively associated with the IC50 of sorafenib in HCC cell lines and Sestrin 2 could induce sorafenib primary resistance by activation of the AKT and AMPK pathways (Dai et al., 2018). 3) Yap, a transcriptional coactivator, is a primary nuclear effector of the Hippo signaling pathway (Sadek and Olson, 2020). Activation of the Hippo/Yap pathway occurs early in the development of liver cancer. Dysregulation of this pathway was detected in approximately 65% of HCCs and associated with a much worse prognosis (Lee et al., 2018). Accumulating studies suggest that Yap play a critical role in sorafenib resistance. For example, Wu et al. found that YAP, activating by SET domain containing 1A (SETD1A), could lead to sorafenib primary resistance in HCC (Wu et al., 2020). 4) FcRn (neonatal Fc receptor) is a member of a family of receptors for the Fc portion of IgG and is involved in the recycling and endocytosis of IgG (Dalloneau et al., 2016). FcRn regulates albumin homeostasis in the liver, where albumin is synthesized (Kuo et al., 2010). Guan et al. found that activation of FcRn triggered the primary resistance of sorafenib in HCC by activating the HIF pathway (Guan et al., 2021). 5) AFP (Alpha fetoprotein) is the most commonly used biomarker for HCC (Zhang et al., 2018). It has been shown that high level of AFP is associated with poor prognosis across stages of HCC (Kelley et al., 2020). Negri et al. confirmed that the adverse prognostic implications of elevated baseline serum AFP and suggested that AFP contributed to the development of sorafenib primary resistance (Negri et al., 2021). 6) CSCs (cancer stem cells) are a subpopulation of cells that have stem cell characteristics and are in an embryonic stem cell state (Wang G et al., 2020). A growing body of research has revealed the critical role of CSCs in intratumoral heterogeneity and primary resistance (Reya et al., 2001). IL-6/STAT3 signaling was shown to be a major pathway to regulate CSCs leading to sorafenib resistance in HCC (Li D et al., 2020). CD133 promotes CSC-like properties by stimulating EGFR-AKT signaling and further reduces the sensitivity to sorafenib in HCC (Jang et al., 2017).

### 2.2 Acquired resistance

Compared with primary resistance, mechanisms for acquired resistance have been widely discussed. Understanding the mechanisms of acquired resistance often has vital therapeutic implications. 1) ABC transporters. Overexpression of ATP binding box (ABC) transporters is a major cause of multidrug resistance (MDR) (Wu and Fu, 2018) For example, Heme oxygenase 1 (HMOX1) reduces the sensitivity of HCC cells to sorafenib via regulation of the expression of ABC transporters (Zhu et al., 2022). In addition, SOX9 contributes to the resistance of HCC to sorafenib by activation of the Akt/ABCG2 pathway (Wang M et al., 2020). 2) Autophagy. Deregulated autophagy is associated with cancer initiation and progression. A large amount of evidence shows that autophagy is involved in developing sorafenib resistance in HCC through various mechanisms, and inhibition of autophagy restores sorafenib sensitivity. For example, CD24 contributes to sorafenib resistance via activating autophagy in HCC. In addition, through FOXO3-mediated autophagy, RNAm 6) A methylation leads to sorafenib resistance in HCC (Lu et al., 2018; Lin H et al., 2020; Li et al., 2021). 3) Exosomes. Exosomes, as material transport carriers, play a vital role in the exchange of biological information and the regulation of the cellular microenvironment (Jiang et al., 2019). Qu et al. showed that HCC cell-derived exosomes induced sorafenib resistance both *in vivo* and *in vitro* via the HGF/c-Met/Akt pathway in HCC (Qu et al., 2016). 4) Ferroptosis. Ferroptosis is an iron-dependent type of non-apoptotic cell death (Youssef et al., 2018). Sorafenib can induce ferroptosis in HCC (Nie et al., 2018). Therefore, inhibition of ferroptosis may induce sorafenib resistance. For example, YAP/TAZ and ATF4 trigger sorafenib resistance by preventing ferroptosis in HCC (Gao et al., 2021b). In addition, Metallothionein-1G leads to sorafenib resistance by inhibition of ferroptosis (Sun et al., 2016). 5) EMT. A number of investigations have shown that EMT is associated with poor survival in patients with HCC because it facilitates tumor development and progression through driving metastasis (Fukusumi et al., 2018). Moreover, EMT is a significant contributor to sorafenib resistance in HCC. For example, Van Malenstein et al. found that continuing exposure to sorafenib of HCC cells triggered the resistance with EMT (van Malenstein et al., 2013). In addition, EMT induced from overexpression of Snails facilitates sorafenib resistance in HCC (Dong et al., 2017). 6) lncRNAs. Long non-coding RNAs (lncRNAs) are a class of non-coding RNAs that participate in an extensive range of biological processes, including cellular proliferation, differentiation and development, exerting a significant influence on normal physiology and disease development (Rodriguez-Mateo et al., 2017). Accumulating studies have shown that some lncRNAs are dysregulated and significantly associated with initiation, metastasis, recurrence, prognosis and drug resistance in HCC (Wei L et al., 2019). Recent studies have confirmed that some lncRNAs participate in sorafenib resistance in HCC. For example, overexpression of LncRNA SNHG1 leads to sorafenib resistance by activation of the Akt pathway (Li et al., 2019). Further, LncRNA NIFK-AS1 was shown to promote sorafenib resistance by m6A methylation in HCC (Chen et al., 2021). Another lncRNA, SNHG3, induces sorafenib resistance by regulating the miR-128/CD151 pathway in HCC (Zhang et al., 2019). 7) MicroRNAs. MicroRNAs (miRNAs) are endogenous small non-coding RNAs consisting of 19–23 nucleotides that regulate eukaryotic gene expression (Asano et al., 2019). Recently, the aberrant regulation of microRNA has been reported to associate with hepatocarcinogenesis and HCC progression (Feng L et al., 2020; Zha and Li, 2020). It was also identified that a variety of miRNAs were involved in sorafenib resistance. For instance, miR-181a contributes to sorafenib resistance by downregulating RASSF1 expression (Azumi et al., 2016). In addition, over-expressed miR-221 leads to sorafenib resistance by inhibiting caspase-3-mediated apoptosis in HCC (Fornari et al., 2017). 8) TME. Development and progression of HCC are a complex process and rely on interactions between the HCC cell and TME. It is generally accepted that TME is linked to aggressive tumor behavior, drug resistance and poor prognosis for cancer patients. Emerging evidence has revealed that various parts of the TME play pivotal roles in sorafenib resistance in HCC (Tang W et al., 2020; Ju et al., 2021). However, these are not the only mechanisms of primary and secondary resistance of sorafenib; the other causes were also being investigated.

## 3 Tumor microenvironment

**FIGURE 2:**
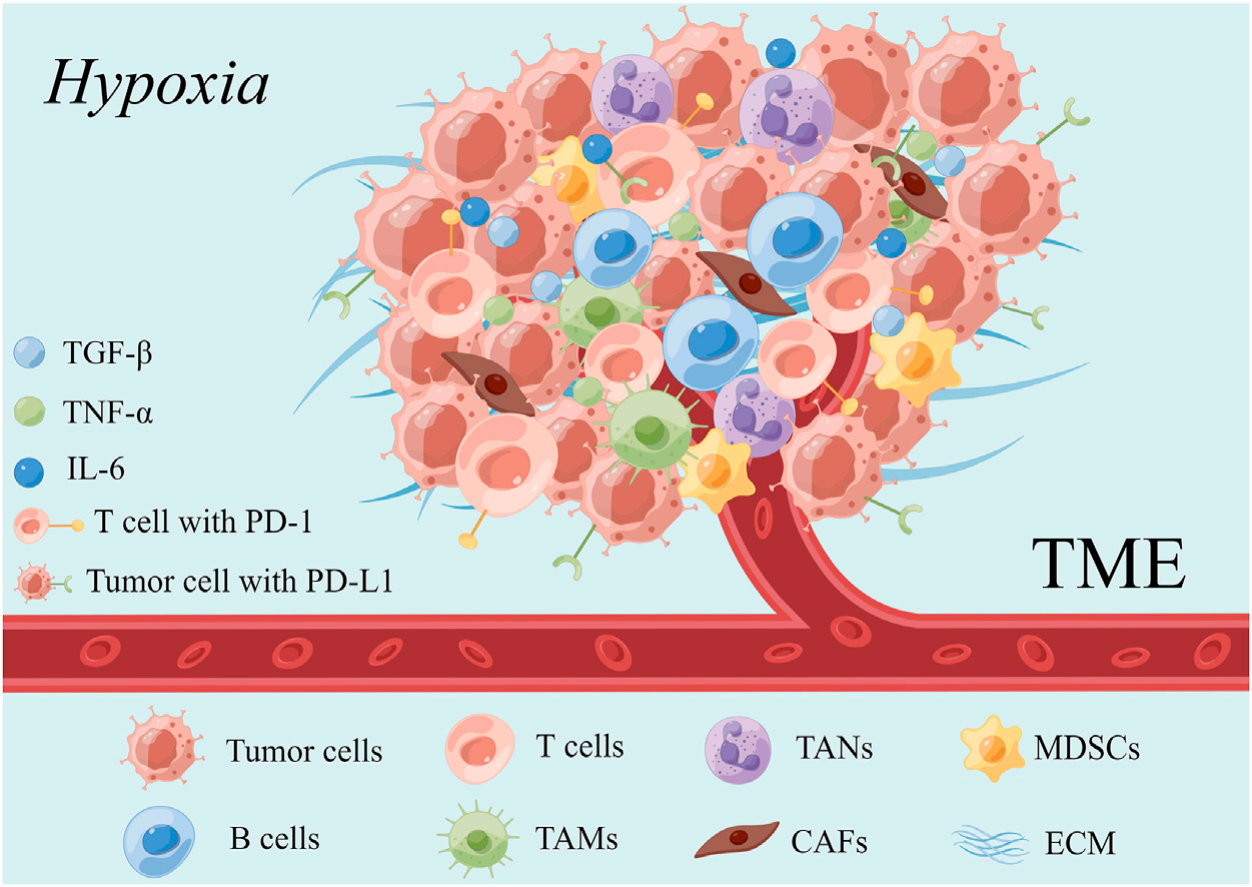
Constituents of the TME (edited by Figdraw software).

TME is a unique internal environment for tumor cells to survive and proliferate. It is a complex network composed of tumor cells, various immune cells, extracellular matrix (ECM) and a variety of cytokines and chemokines (Mao et al., 2021). The composition of the TME is shown in Figure 2. Studies have highlighted that TME tends to be a hypoxic and acidic environment, which can affect the tumor phenotype and promotes the metastasis and proliferation of tumor cells (Abou Khouzam et al., 2022).

In general, TME is infiltrated with many tumor-related immune cells, including anti-tumor immune cells, such as CD8^+^ cytotoxic T lymphocytes (CTL) and natural killer cells (NK), and immunosuppressive cells. However, these immune cells are presented at a weakened or non-functional state (Binnewies et al., 2018). A large number of CTLs are limited to the border of tumor mass or segmented by fibrotic nests and are exhibited to hamper the killing activity of tumor cells (Binnewies et al., 2018). Meanwhile, regulatory T cells (Treg), myeloid-derived suppressor cells (MDSCs), and tumor-associated macrophages (TAMs) are activated and proliferate in large numbers in TME to inhibit the function of anti-tumor effector cells and promote the immune escape and metastasis of tumor cells (Khalaf et al., 2021).

Emerging investigations have demonstrated that ECM, a complex network composed of protein crosslinks, provides physical support for cell growth within TME. Hyaluronic acid and collagen are the major compositions of ECM, which increase solid stress and interstitial fluid pressure, compress the tumor vascular system, and participate in tumor growth, angiogenesis, immunosuppression, and chemoradiotherapy resistance (Zhang et al., 2022). Interestingly, ECM deposition was shown to generate “fibrotic nests” that enclose CTLs to a poor immunological state, forming a barrier that prevents CTLs infiltration into the tumor core (Binnewies et al., 2018).

In TME, cancer-associated fibroblasts (CAFs), the most abundant cells, are able to interact with adjacent tumor cells mediating by paracrine signals (cytokines, exosomes, and metabolites) or the ECM. Moreover, CAFs secret matrixmetallo proteinases (MMPs) to reshape the ECM. Moreover, by releasing chemokines, growth factors, and angiogenic factors, they also contribute to the abnormalities and functional defects of vascular structure, thereby accelerating tumor invasion and metastasis and promoting the occurrence of drug resistance (Mao et al., 2021). A recent study found that the exosomes released by gemcitabine-exposed CAFs could more effectively accelerate of tumor cell growth (Richards et al., 2017), suggesting the potential role of CAFs in drug resistance.

In addition, the interactions between cytokines released from the local TME and their receptors make the tumor form an immunosuppressive network, which promotes tumor progression. Several studies have demonstrated that interleukin-6 (IL-6), transforming growth factor-β (TGF-β), TNF-α, CCL2, CCL17, CCL22, and other immune-regulatory cytokines have significant changes in TME and are closely related to tumor grade, invasiveness and sorafenib resistance.

Until now, it has been well established that TME is a complex and dynamic network driving tumor growth and progression. Although regulated by the combined actions of many factors, TME maintains relative stability. Of note, TME-mediated drug resistance is usually the result of the continuous interactions between tumor cells and components of the TME. In this way, targeting various parts of the tumor microenvironment could be one of the effective strategies to overcome the drug resistance.

## 4 Relationships between sorafenib resistance and tumor microenvironment

### 4.1 Hypoxia

As a common pathophysiological phenomenon, hypoxia is present in most solid tumors, including HCC (Curtis et al., 2019) (Chiu et al., 2017). The median partial pressure of O2 (pO2) is barely 6 mmHg in human liver tumors. However, in normal human liver tissue, the pO_2_ is 30 mm Hg (Bao and Wong, 2021). Tumors have developed a variety of mechanisms to cope with hypoxic stress. Among the various regulatory pathways, hypoxia-inducible factors (HIFs) are the most important transcription factors that regulate a couple of dozen genes in response to a decrease in intracellular oxygen concentration (Wang and Liu, 2020). HIFs are heterodimers composed of a HIF-α subunit (HIF-1α, HIF-2α, or HIF-3α) and a HIF-1β subunit (Bulle and Lim, 2020). As a negative regulator of HIF-mediated gene expression, HIF-3α is not closely related to HIF-1α and HIF-2α (Zou et al., 2011). Therefore, we will primarily discuss HIF-1α and HIF-2α. The HIF-α subunits are tightly regulated by cellular oxygen concentration, whereas the HIFβ-subunit is persistently expressed (Lee et al., 2020). Under normoxic condition, HIF-1α and HIF-2α are catalyzed to be degraded by von Hippel-Lindau tumor suppressor protein (VHL) and cannot activate the transcription of their target genes (Shi et al., 2009). However, HIF-1α and HIF-2α are stabilized under hypoxic conditions leading to transcriptionally inducing the target genes involved in energy metabolism, angiogenesis, proliferation, apoptosis and drug resistance (Shih et al., 2017; Vanhove et al., 2019).

**FIGURE 3:**
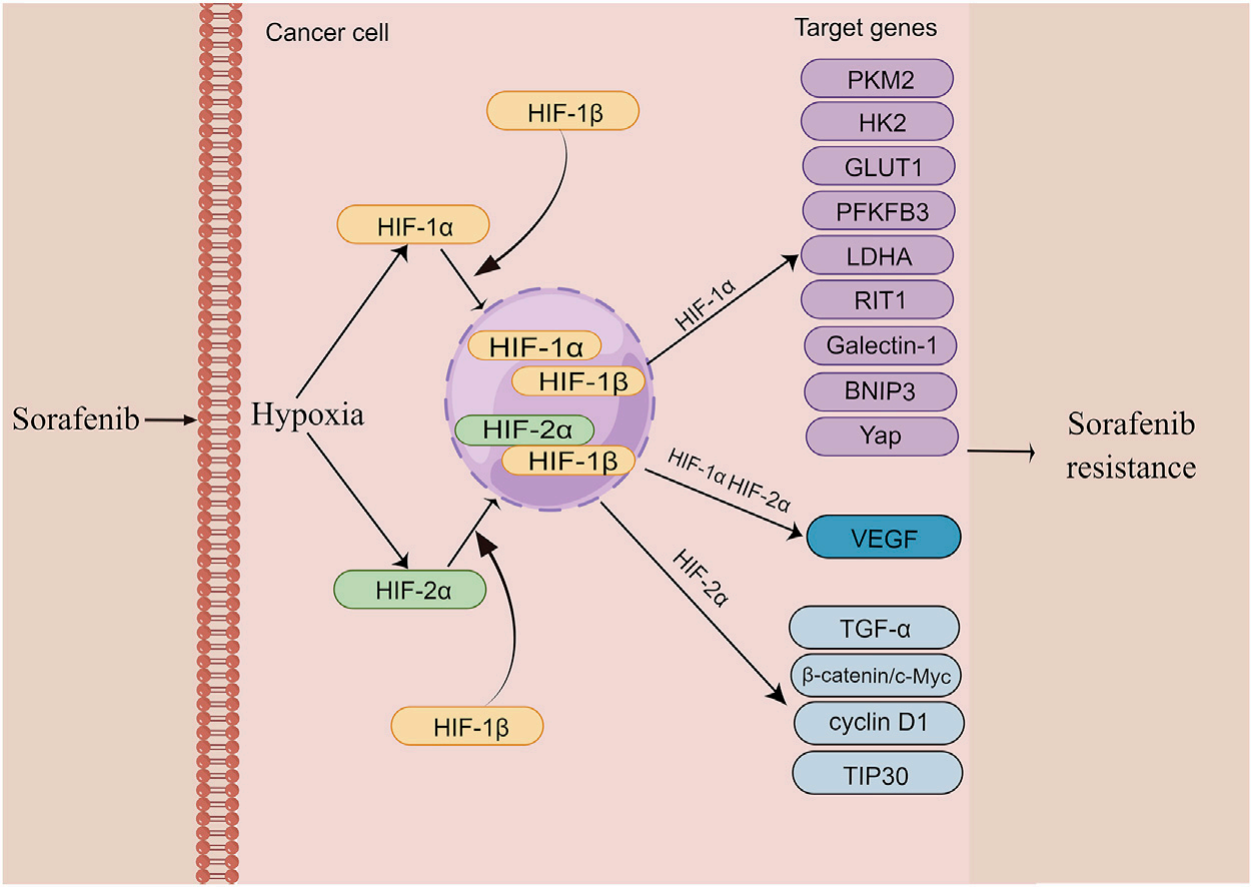
The mechanism of hypoxia induces sorafenib resistance in HCC (edited by Figdraw software). Sustained sorafenib treatment induces dysregulation of HIF-1a and HIF-2a expression and promotes transcription of their downstream genes, thereby causing resistance to sorafenib in HCC.

Increasing evidence indicates that hypoxia plays a prominent role in the drug resistance, including sorafenib resistance in various cancers and different therapies resistance in HCC (Alsaab et al., 2018; Wu et al., 2019). Compared with sorafenib-sensitive patients, sorafenib-resistant tumors typically show higher intratumoral hypoxia (Liang et al., 2013). It is worth noting that long-term sorafenib treatment also exacerbates the hypoxic microenvironment of HCC by suppressing tumor angiogenesis (Mo et al., 2021). Sorafenib-induced hypoxia stabilizes HIF-1α and HIF-2α and strengthens the transcription of their downstream target genes (Song et al., 2019). This process acts as an adaptive cytoprotective response to induce sorafenib resistance in liver cancer cells. In addition, sorafenib also triggers the HIF-1α-to-Hif-2α pathway switch, further promoting this adaptive cytoprotective response (Ma et al., 2014). The entire process can enhance the effect via positive feedback loop and form a vicious cycle, accelerating the resistance to sorafenib. Accordingly, inhibition of hypoxia is a promising strategy for overcoming sorafenib resistance. In this section, we mainly discuss the relationship between hypoxia and sorafenib resistance in HCC. The mechanism details are described in Figure 3.

#### 4.1.1 HIF-1α

HIF-1a is frequently upregulated in patients with HCC, and its overexpression is largely related to poor prognosis of HCC patients (Kai et al., 2016). An increasing body of evidence suggests that metabolic alterations in the glycolytic pathway play an essential role in drug resistance (Mondal et al., 2019). Glycolysis-mediated drug resistance is frequently associated with the upregulation of glycolysis-related vital enzymes, including pyruvate kinase type M2 (PKM2), Hexokinase II (HK2), glucose transporter 1 (GLUT1), and PFKFB3 (Xia et al., 2020). Indeed, HIF-1a occupies a significant position in regulating these glycolysis enzymes. For example, the activity of PKM2, which is highly upregulated and initiates sorafenib resistance in HCC, is mediated by HIF-1a (Chen J et al., 2018) In contrast, Simvastatin, a medicine to lower lipid level, can overcome sorafenib resistance by inhibiting HIF-1α/PPAR-γ/PKM2-mediated glycolysis (Feng J et al., 2020). Another drug flavonoid proanthocyanidin B2 was shown to enhance the efficiency of sorafenib by targeting PKM2 (Feng et al., 2019). HK2, which catalyzes glucose to glucose 6-phosphate (G6P) in the glycolytic pathway, is negatively related to poor prognosis in patients with HCC and is also mediated by HIF-1a (Agnihotri and Zadeh, 2016; DeWaal et al., 2018). Compared with the responders, sorafenib-resistant patients showed an elevated level of HK2 (Gao et al., 2021b). Whereas HK2 knockdown synergistically inhibited tumor growth with sorafenib, suggesting that HK2 inhibition significantly improves the efficacy of sorafenib (DeWaal et al., 2018). In addition, HIF-1α was shown to reduce the expression of miR-199a that directly targets PKM2 and HK2 in liver cancer (Guo et al., 2015). As a HIF target gene, GLUT1 expression is associated with resistance to multiple drugs in various cancers (Chigaev, 2015; Sawayama et al., 2019; Guo et al., 2020). It has been demonstrated that genistein inhibited HK2 and GLUT1 to suppress aerobic glycolysis and improve sorafenib sensitivity by downregulation of HIF-1α (Li et al., 2017). PFKFB3 is a member of the 6-phosphofructo-2-kinase/fructose-2,6-bisphosphatases (PFKFB) family and is closely related to many aspects of cancer including cell proliferation, vessel aggressiveness, drug resistance and TME (Shi et al., 2017). The expression of PFKFB3 is markedly induced under hypoxia condition (Niskanen et al., 2018; Sun et al., 2019). Meanwhile, PFKFB3 was found to be elevated after sorafenib treatment and the increased PFKFB3 markedly hampered sorafenib sensitivity in HCC cells. Interestingly, the inhibition of HIF-1α overcomes sorafenib resistance by modulating PFKFB3 in HCC (Long et al., 2019). Ras-like-without-CAAX-1 (RIT1), a member of the Ras family of GTPases, has emerged as an important cause of Noonan syndrome and cancer (Castel et al., 2019). It has been revealed that hypoxia significantly upregulates RIT1 expression in HCC cells via HIF-1α and the over-expressed RIT1 attributes to sorafenib resistance in HCC (Song et al., 2019). A study found that RIT1 was able to promote cell proliferation by activation of AKT. As a result, the combination of sorafenib and AKT inhibitor enhances sorafenib sensitivity in HCC (Sun et al., 2022). Bcl-2 interacting protein 3 (BNIP3), a member of the BH3-only Bcl-2 family, is a hypoxia-regulated protein. HIF-1α increases BNIP3 expression by binding to a hypoxia response element (HRE) within the promoter region of BNIP3 (Burton et al., 2006). The methylation BNIP3 promoter was observed in sorafenib resistant HCC cells under hypoxia (Mendez-Blanco et al., 2019). Galectin-1, belonging to the galectin protein family, is suggested to be a predictive marker of poor prognosis and a potential therapeutic target for malignant tumors (Wu et al., 2012). As a downstream target of the AKT/mTOR/HIF-1α signaling pathway, Galectin-1 is a possible biomarker for predicting resistance of sorafenib in HCC *in vitro* and *in vivo* (Yeh et al., 2015). Furthermore, Galectin-1 was shown to induce sorafenib resistance in liver cancer by activation of the FAK/PI3K/AKT signaling (Zhang P.F et al., 2016). YAP is activated and translocated into the nucleus under hypoxia (Li et al., 2018). It has been reported that the Hippo/YAP/TAZ pathway is involved in drug resistance, cancer cell stemness and EMT (Gao et al., 2021b). Of note, YAP/TAZ drives sorafenib resistance in HCC by preventing ferroptosis (Gao et al., 2021b). YAP promotes sorafenib resistance in HCC by inducing survival as well (Sun et al., 2021). Furthermore, cirrhotic stiffness induces sorafenib resistance in HCC via YAP (Gao J et al., 2019). Additionally, it has been shown that YAP–IGF1R signaling plays a vital role in sorafenib resistance and targeting YAP–IGF-1R is an effective measure for treating sorafenib-resistant HCC (Ngo et al., 2021). Due to the crucial role of HIF-1a in regulating sorafenib resistance, previous studies have demonstrated that a dozen of drugs improve sorafenib resistance in HCC by indirect targeting HIF-1a. For instance, melatonin reduces HIF-1α protein synthesis by inhibiting the mTORC1/p70S6K/RP-S6 pathway, thereby improving sorafenib sensitivity (Prieto-Dominguez et al., 2017). *Rhizoma Paridis* saponins extracted from the herb *Paris polyphylla* decreases mRNA and protein levels of HIF- 1a and the combination with sorafenib reduces the resistance (Yao et al., 2018). In summary, a deeper understanding of HIF-1a in sorafenib resistance provides a potential therapeutic target for overcoming the resistance. In addition, glycolysis-related pathways seem to be the central element, and further investigation is warranted for metabolomics in sorafenib resistance.

#### 4.1.2 HIF-2a

HIF-1α and HIF-2α have been suggested to exist the reciprocal compensatory mechanism, by which the expression of HIF-2α can be upregulated when HIF-1α is inhibited (Menrad et al., 2010). This switch is conducive to generate dynamic cytokines for tumors’ aggressive growth under hypoxia (Koh et al., 2011). Similar to HIF-1α, the expression of HIF-2α is also induced by sorafenib, leading to the insensitivity to sorafenib in HCC cells (Zhao et al., 2014). As such, the re-sensitization of the resistant HCC cells to sorafenib can be improved by regulating HIF-2α and its downstream genes. For example, Ma et al. showed that sorafenib-induced upregulation of HIF-2α and increased expression of vascular endothelial growth factor (VEGF) and cyclin D1 contribute to the resistance of hypoxic HCC cells to sorafenib. Both HIF-1α and HIF-2α, as well as their downstream genes, including VEGF, lactate dehydrogenase A (LDHA) and cyclin D1, are significantly reduced by 2ME2, an antitumor and antiangiogenic agent (Ma et al., 2014). In addition, sodium orthovanadate also overcomes sorafenib resistance in HCC cells by reduction of HIF-1α/HIF-2α protein expression and their nuclear translocation, resulting in downregulation of their downstream genes, including VEGF, LDHA and GLUT1 (Jiang et al., 2018). Interestingly, sorafenib treatment-upregulated HIF-2α by sorafenib feedback enhances sorafenib resistance by activating the TGF-α/EGFR pathway (Zhao et al., 2014). HIF-2α activity mediated by the COX2/PGE2 axis was found to be associated with the activation of TGF-α/EGFR, which in turn promotes HCC development and reduces the sensitivity to sorafenib. The β-catenin/c-Myc pathway is an essential signaling pathway in tumors (Bai et al., 2020). Liu et al. found that activation of β-catenin/c-Myc signaling enhances glycolysis and glutaminolysis, and promotes hepatocarcinogenesis, metastasis, and drug resistance (Liu et al., 2019). Notably, downregulation of HIF-2α improves the antitumor activity of sorafenib in HCC via the β-catenin/C-Myc-dependent pathway (Liu et al., 2015). A 30-kDa Tat-interacting protein (TIP30), a tumor suppressor gene and a downstream target of HIF-2α, was shown to inhibit EMT. TIP30 downregulated by the overexpression of HIF-2α has been identified to result in EMT (Zhu et al., 2015). Surprisingly, metformin was found to enhance the anti-tumor activity of sorafenib by regulation the expression of HIF-2α and TIP30 (You et al., 2016).

### 4.2 Tumor-associated immune-suppressive cells

**FIGURE 4:**
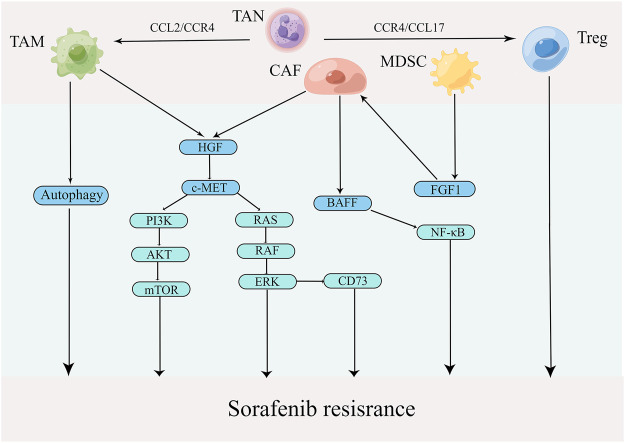
Mechanisms by which tumor-associated immune-suppressive cells promote sorafenib resistance in TME (edited by Figdraw software).

The extensive infiltration of tumor-associated immune-suppressive cells in TME is considered a principal factor affecting cancer progression and hindering treatment (Gorgun et al., 2013). When tumor-associated immunosuppressive cells are recruited into TME, they promote the malignant phenotypes of HCC (Riscal et al., 2019). In addition, these immunosuppressive cells establish a complex of interaction network that maintains the immunosuppressive microenvironment and promotes the immune escape of tumor cells (LaGory and Giaccia, 2016). A growing body of literature has recently shown that the infiltration of tumor-associated immunosuppressive cells is a vital link in sorafenib resistance. Therefore, clarification of the relationship between tumor-associated immunosuppressive cells and sorafenib resistance is crucial. The mechanisms of tumor-associated immunosuppressive cells contributing to sorafenib resistance are presented in Figure 4.

#### 4.2.1 Tumor-associated macrophages

TAMs, whose functions are determined by their polarization state, are one of the most abundant types of immune cells in TME (Le et al., 2018). Polarized macrophages have been identified into two broad types: M1 (classically activated macrophages) and M2 (alternatively activated macrophages). TAMs frequently convert M1 to M2 during tumor progression, supporting tumor growth and metastasis by various functions (Zhu Z et al., 2020). Mantovani et al. found that abundant TAMs are associated with poor prognosis in various cancers, especially liver cancer (Mantovani et al., 2006). Furthermore, TAMs are closely related to sorafenib resistance as well. For example, Wei et al. demonstrated that TAMs promoted proliferation, migration and invasion of sorafenib-resistant liver cancer cells (Wei et al., 2017). A recent study showed that TAMs mediated liver cancer resistance to sorafenib by activating the MAPK, PI3K/AKT and HGF/c-Met signaling pathways (Dong et al., 2019). Additionally, a growing number of studies have shown that autophagy that was induced by M2 macrophages is a significant causing factor of sorafenib resistance in HCC (Prieto-Dominguez et al., 2016; Wu et al., 2016; Lin Z et al., 2020). For example, Wei et al. found that M2 macrophages boost autophagy when sorafenib acts on tumor cells; however, this autophagy renders tumor cells resistance to sorafenib (Wei F et al., 2019). Compared with traditional treatments, targeting macrophages has become a new strategy in cancer immunotherapy (Cassetta and Kitamura, 2018; Gunassekaran et al., 2021). The photoimmunotherapy utilizing a TAM-targeted probe IRD-αCD206 was found to suppress the growth and metastasis of sorafenib-resistant tumor (Zhang C et al., 2016). In addition to eliminating TAM cells, repolarizing TAM from M2 to M1 phenotype is another promising intervention approach in cancer (Snuderl et al., 2013). For example, IFN-α, as an immunomodulator, was shown to increase the therapeutic efficacy of sorafenib via a shift in TAM polarization (Zhang et al., 2021). In addition, the compound Kushen, the dried roots of Sophora flavescens Aiton, injection was found to induce polarization TAMs to M1 and thus reverse sorafenib resistance (Yang Y. et al., 2020). Notably, CCL2, a member of the C-C chemokine family, promotes the recruitment of TAMs by activating CCR2, leading to cancer progression (Yang Z et al., 2019). CCR2 antagonist, 747, was able to block TAM recruitment and enhance the efficiency of sorafenib by modulating the CCL2/CCR2 axis, providing a novel therapeutic approach for HCC (Yao et al., 2017). These results indicate the importance of TAMs in sorafenib resistance in HCC.

#### 4.2.2 Cancer-associated fibroblasts

A large number of CAFs in the tumor tissue create a favorable environment for tumor development (Pein et al., 2020). CAFs not only boost tumor growth and metastasis but also mediate immunosuppression and drug resistance by directly interacting with cancer cells or by secreting a wide variety of factors and nutrients (Multhaupt et al., 2016). Mechanistically, CAFs lead to the resistance by impairing drug delivery and biochemical signaling (Meads et al., 2009). In addition, ECM remodeling by CAFs inhibits anti-cancer drug uptake through increasing intratumoral interstitial fluid pressure and vascular collapse (Paraiso and Smalley, 2013). Liu et al. showed that the co-culture of liver tumor organoids with CAFs could decrease the efficiency of sorafenib, 5-FU and regorafenib (Liu et al., 2021). It has also been demonstrated that CAFs induce sorafenib resistance by activation of the BAFF/NF-κB axis in liver cancer cells (Gao L et al., 2021). Another report demonstrated that HGF secreted by CAFs regulates the expression of CD73 to promote the sorafenib resistance of HCC by modulating the Met-ERK1/2 pathway (Peng et al., 2020). Therefore, CAF is of crucial role in sorafenib resistance and could be a potential immunotherapeutic target for overriding the resistance.

#### 4.2.3 Tumor-associated neutrophils

Emerging evidence indicates that neutrophils, which have been identified to regulate innate and adaptive immune responses, also play essential roles in sorafenib resistance (Gupta and Kaplan, 2016). It has been reported that neutrophils display plasticity. Similar to TAMs, TANs can either be polarized into an anti-tumorigenic “N1” phenotype by IFNs or into a protumorigenic “N2” phenotype when TGF-β is present (Strauss et al., 2021). In clinical trials, enriched N2 TANs in HCC tumor tissues are not only a poor prognostic marker but also a key indicator of the poor efficacy of sorafenib in patients with HCC (Li et al., 2011; Bruix et al., 2017). Clinical data have also shown that sorafenib is more effective in treating patients with less N2 TANs infiltration (Zhou et al., 2016). Hence, the overrepresentated N2 TANs is closely related to sorafenib resistance in liver cancer. Further study has highlighted that N2 TANs recruit macrophages and Treg to promote resistance to sorafenib and the progression of liver cancer (Zhou et al., 2016). Future investigations are required to develop strategy aiming to suppress the recruitment of immunosuppressive cells including N2 TANs for conquering sorafenib resistance.

#### 4.2.4 Myeloid-derived suppressor cells

MDSCs are a heterogeneous cell population comprising of progenitors and precursors of myeloid cells with potent immunosuppressive effect (Mulder et al., 2019). There are three major types of MDSCs: polymorphonuclear MDSCs (PMN-MDSCs), monocytic MDSCs (M-MDSCs) and early-stage MDSCs (eMDSCs). Phenotypically and morphologically, PMN-MDSCs resemble neutrophils, while M-MDSCs are similar to monocytes. eMDSCs are primarily myeloid progenitors and precursors, and represent less than 5% of MDSCs (Veglia et al., 2018; Veglia et al., 2021). Although the phenotypic characteristics of PNM-MDSCs and M-MDSCs differ, both possess potent immunosuppressive properties. MDSCs promote the development of liver cancer through a variety of mechanisms, including inhibition of CD8^+^ T-cell response, induction of Treg expansion and impairment of NK cell function (Hoechst et al., 2009; Kalathil et al., 2013; de Oliveira et al., 2016). In addition, MDSCs are associated with early recurrence and poor prognosis in HCC patients who have undergone curative resection, radiotherapy and hepatic arterial infusion chemotherapy (Deng et al., 2022). Deng et al. found that MDSCs facilitated CAF activation, resulting in tumor growth, angiogenesis and sorafenib resistance by inducing FGF1 expression (Deng et al., 2022).

#### 4.2.5 Regulatory T cells

Treg cells are defined as a FoxP3+ CD25^+^ CD4^+^ T lymphocyte subset (Konopacki et al., 2019). They protect hosts from developing autoimmune diseases and allergies, whereas in malignancies, they promote tumor progression by suppressing effective antitumor immunity. FoxP3+ Treg is a potential therapeutic target to enhance the effect of antitumor immunity (Whiteside, 2018). Gao et al. found that intratumoral CCR4+ Tregs were the leading type of Tregs and closely associated with sorafenib resistance in hepatitis B-related HCC. Moreover, interfering with a CCR4 antagonist or a N-terminus recombinant protein of CCR4 (N-CCR4-Fc) exerts a prominent effect on conquering sorafenib resistance and sensitizes liver cancer to PD-1 checkpoint inhibitor (Gao et al., 2022). It is of considerable interest to identify new approaches that target Tregs for overcoming sorafenib resistance in HCC.

### 4.3 Immunosuppressive factors

Numerous studies have revealed that some immunosuppressive factors, that were secreted by tumor cells or stromal cells, infiltrate into the tumor site through specific recognition. These immunosuppressive molecules, together with other components of the TME, form a stable immunosuppressive microenvironment to mediate the immune escape of tumor cells and sorafenib resistance. The mechanisms of these factors in sorafenib resistance are shown in Table 1.

**TABLE 1:** Immunosuppressive factors and sorafenib resistance in HCC.

Immunosuppressive factor	Cell involved	Pathway	Effects on the tumor	References
PD-L1	HepG2 and Huh7 sorafenib-resistance cells	PD-L1/STAT3/DNMT1	Promote sorafenib resistance	Liu et al., (2017)
	Huh7 sorafenib-resistance cells	c-MET/PD-L1/RAS/RAF/MEK/ERK1/2	Promote sorafenib resistance	Xu et al., (2022)
	HepG2 and Huh7 sorafenib-resistance cells	PI3K/AKT/SREBP-1	Promote sorafenib resistance	Xu et al., (2020)
TGF-β	Hep3B and PLC/PRF/5 cells	TGF-β/EMT/PD-L1	Promote sorafenib resistance	Shrestha et al., (2021b)
	Hep-3B, Huh7, SK-Hep-1, SNU-182, SNU-398 and SNU-449 cells	TGF-β/P38	Inhibit sorafenib resistance	Kang et al., (2017)
	HepG2, Huh7 and PLC/PRF/5 cells	TGF-β/ERK/ETS1/PXR	Promote sorafenib resistance	Bhagyaraj et al., (2019)
	PLC/PRF/5, Hep3B and Huh7 cells	TGF-β/RTK	Promote sorafenib resistance	Ungerleider et al., (2017)
IL-6	PLC/PRF/5 sorafenib-resistance cells	LCSCs/IL-6/STAT3	Promote sorafenib resistance	Li Y et al., (2020)
	Hep3B, HepG2, Huh7 and HepG2.2.15 Hep3B and HepG2.2.15 sorafenib-resistance cells	IL-6/STAT3/DNMT3b/OCT4	Promote sorafenib resistance	Lai S.C et al., (2019)
	HEK-293T, Huh7 and Hep3B cells Huh7 and Hep3B sorafenib-resistance cells	PSMD10/IL-6/STAT3/DANCR	Promote sorafenib resistance	Liu et al., (2020)
CCL2	HepG2, PLC/PRF/5, MHCC97H, HCCLM3, Hepa1-6 and H22cells	CCL2/CCR4/TAM	Promote sorafenib resistance	Zhou et al., (2016)
CCL17	HepG2, PLC/PRF/5, MHCC97H, HCCLM3, Hepa1-6 and H22cells	CCL17/CCR4/Treg	Promote sorafenib resistance	Zhou et al., (2016)
CXCR3	Huh7 sorafenib-resistant cells	CXCR3/MAPK pathway/adipocytokine signaling	Promote sorafenib resistance	Ren et al., (2020)
SDF-1α	HCA-1 cells	SDF-1α/CXCR4	Promote sorafenib resistance	Chen et al., (2014)

#### 4.3.1 PD-L1

Programmed cell death 1-ligand 1 (PD-L1), also known as B7-H1 or CD274, is one of the most critical immune inhibitory molecules in the TME and plays a vital role in tumor cell immune escape (Theodoraki et al., 2018). It has been widely reported that PD-L1 regulates drug resistance and other malignant phenotypes in many types of cancer (Nowicki et al., 2018; Shen et al., 2019; Chen et al., 2020). PD-L1 was found to be overexpressed in sorafenib-resistant HCC cell lines and tumor tissues (Zhang et al., 2020). Liu et al. showed that overexpression of DNA methyltransferase1 (DNMT1) is positively associated with elevated level of PD-L1 in sorafenib-resistant HCC cells, and PD-L1 is able to regulate DNMT1 via the STAT3 signaling pathway. Furthermore, the inhibition of either PD-L1 or DNMT1 sensitizes HCC cells to sorafenib (Liu et al., 2017). In addition, c-Met and PD-L1 were shown to be co-overexpressed in sorafenib-resistant cell lines, and c-Met promoted the expression of PD-L1 through the MAPK/NF-κBp65 cascade. The overexpressed PD-L1 in turn facilitates sorafenib resistance (Xu et al., 2022). By activating Sterol regulatory element-binding protein 1 (SREBP-1) via the PI3K/AKT signaling, PD-L1 promoted EMT in sorafenib-resistant HCC cell lines (Xu et al., 2020). Accordingly, inhibiting PD-L1 is an excellent approach to overcome sorafenib resistance. A previous study revealed that microRNA-1 overcame sorafenib resistance and suppressed the malignant progression of liver cancer cells through inhibition of PD-L1 (Li D et al., 2020). Similar to EMT inhibition, silencing PD-L1 was also shown to sensitize cells to sorafenib (Shrestha et al., 2021b). Strikingly, it has been reported that combination with EMT inhibition, blockade of PD-L1 expression exhibits potent effects on sorafenib resistance by targeting the liver cancer stem cell subpopulation (Shrestha et al., 2021a). Clinical studies have also provided evidence that avelumab, an anti-PD-L1 antibody, shows moderate efficacy and is well tolerated in advanced HCC patients previously treated with sorafenib (Lee et al., 2021). Based on these findings, the combination of PD-L1 inhibitor with sorafenib could be an effective therapeutic strategy for advanced HCC.

#### 4.3.2 TGF-β

It is well known that transforming growth factor-β (TGF-β) exerts dual effects on tumor cells, both positive and negative functions (Massague, 2008). In the early stage of cancer development, TGF-β acts as a tumor suppressor to inhibit cell proliferation and stimulate apoptosis. However, in the late stage of cancer development, TGF-β becomes a tumor-promoting factor to induce EMT, invasion and metastasis. In addition, TGF-β is a key regulator of T cell response. It also regulates the responses mediated by the innate and adaptive immune cells, including dendritic cells, B cells, NK cells, innate lymphocytes, and granulocytes (Tu et al., 2019). In bioinformatic studies, Lin et al. found that mRNA levels of TGF-β were elevated in sorafenib-acquired resistant HCC tissues (Lin H et al., 2020). Numerous studies have shown that overexpressed TGF-β is associated with HCC progression and sorafenib resistance (Lin et al., 2015; Sharma et al., 2017). Bhagyaraj et al. found that TGF-β increased pregnane X receptor (PXR) expression via the ERK-ETS1 axis and contributed to sorafenib resistance (Bhagyaraj et al., 2019). In addition, TGF-β induces the expression of receptor tyrosine kinases (RTKs) that contribute to sorafenib resistance in HCC (Ungerleider et al., 2017). Therefore, TGF-β plays a crucial role in the process of sorafenib resistance. As EMT is a critical process of drug resistance and as TGF-β is a major signal transduction pathway in EMT (Hahne and Valeri, 2018; Kouno et al., 2019), the sorafenib resistance induced by TGF-β could be closely related to EMT. In addition, Shrestha et al. revealed that TGF-β1-induced EMT increased PD-L1 expression in HCC cells, leading to the resistance of sorafenib. In line with these findings, the combination of targeting PD-L1 and TGF-β1 signals was shown to have a synergic effect on conquering sorafenib resistance (Shrestha et al., 2021b). Another study showed that knockdown of TGF-β could reinforce the phosphorylation of p38 and enhance the sensitivity of HCC cells to sorafenib (Kang et al., 2017). TGF-β has also been shown to induce sorafenib resistance through the ERK/AKT signaling pathway, and valproic acid increases sorafenib sensitivity by suppressing TGF-β-induced ERK/AKT signaling (Matsuda et al., 2014). In addition, miR-101 improves the anti-tumor effect of sorafenib in HCC cells by targeting dual-specificity phosphatase 1 (DUSP1) and inhibiting TGF-β activation (Wei et al., 2015). Consequently, the desensitization of TGF-β is a potential strategy for overcoming sorafenib resistance.

#### 4.3.3 TNF-α

TNF-α, an important pro-inflammatory cytokine, is produced by activated macrophages/monocytes (Yu et al., 2020). Numerous clinical and experimental studies have revealed that patients with liver damage produce large amounts of TNF-α, which are closely related to the incidence and progression of hepatitis, liver cirrhosis and liver cancer (Huang et al., 2017). Liu et al. showed that overexpression of TNF-α was related to poor prognosis in HCC patients (Liu et al., 2013). The elevated level of TNF-α is also associated with the weakened effect of sorafenib treatment (Tan et al., 2019). TNF-α was found to accelerate sorafenib resistance by inducing EMT in HCC cells. Moreover, ulinastatin, an urinary trypsin inhibitor, improves the antitumor effect of sorafenib by inhibition of TNF-α expression and secretion (Tan et al., 2019). A recent study has shown that TNF-α derived from the inflammatory microenvironment of the fibrotic liver promotes sorafenib resistance via STAT3 activation and that STAT3 antagonists reverse HCC resistance to sorafenib (Jiang et al., 2021). Additionally, sorafenib was demonstrated to promote CCL22 expression via the TNF-α/RIP1/NF-κB signaling, and the sorafenib resistance was reversed by inhibition of CCL22 signaling, suggesting CCL22 as a possible therapeutic target for hampering sorafenib resistance (Gao et al., 2020; Marshall et al., 2020).

#### 4.3.4 IL-6

IL-6, a significant driver of hepatocellular carcinogenesis, is involved in tumor progression, metastasis and chemoresistance in HCC (Bharti et al., 2016). A growing body of evidence suggests that HCC cancer stem cells (CSCs) are responsible for the tumor recurrence and sorafenib resistance (Lai Y et al., 2019). Liver cancer stem cells (LCSCs) were shown to accelerate sorafenib resistance via the IL-6/STAT3 signaling pathway, and targeting IL-6 relieves this resistance (Li Y et al., 2020). The IL-6/STAT3 signaling pathway also mediates sorafenib resistance by increasing expression of DNA methyltransferase 3b (DNMT3b) and octamer-binding transcription factor 4 (OCT4). Combination of targeting DNMT3b with nanaomycin A and sorafenib treatment manifested a synergistic inhibitory effect on sorafenib-resistant HCC cells (Lai S.C et al., 2019). In addition, a long-noncoding RNA (lncRNA) DANCR was shown to promote sorafenib resistance by activating the IL-6/STAT3 pathway. Similarly, the activation of the IL-6/STAT3 pathway feedback enhances DANCR expression (Liu et al., 2020). A previous report also demonstrated that celecoxib, an anti-inflammatory medicine, overcame sorafenib resistance by inhibition of the IL-6/STAT3 signaling cascade (Liu et al., 2011). In summary, these findings indicate that the IL-6/STAT3 pathway plays a vital role in promoting sorafenib resistance and could be critical therapeutic targets for defeating the resistance.

#### 4.3.5 Chemokines

Chemokines are a class of cytokines that have similar structures and chemotactic functions. Based on the sequence of cysteine residues at the N-terminus, chemokines are divided into four subtypes: CC-chemokines, CXC-chemokines, C-chemokines and CX3C-chemokines (Affo et al., 2014; Chen W et al., 2018). Chemokines and their receptors, which have been identified to play a critical role in the development and metastasis of cancers, have altered expression in a variety of tumors (Gao et al., 2018; Vela et al., 2019). Recently, an increasing number of studies have reported that CC chemokines and CC receptors (CCRs) are involved in sorafenib resistance. For example, Zhou et al. found that CCL2/CCR2 and CCL17/CCR4 secreted from TANs are responsible for sorafenib resistance by recruiting macrophages and Tregs (Zhou et al., 2016). A CCR2 antagonist, 747, enhances the anti-cancer efficacy of sorafenib by blocking TAMs (Yao et al., 2017). Similar to CCR2, CCR4 is a therapeutic target for sorafenib resistance as well. It has been shown that CCR4 antagonism enhances anti-cancer efficiency of sorafenib and overcomes sorafenib resistance via targeting CCR4+TIL-Tregs (Gao et al., 2022). Sorafenib also increases CCL22 expression by activation of TNF-α/RIP1/NF-κB signaling. In contrast, inhibition of CCL22 surmounts sorafenib resistance (Gao et al., 2020). Furthermore, sorafenib-resistant cells exhibited higher levels of CXC chemokines and CXC-chemokine receptors (CXCRs) (Wu et al., 2017). For example, Ren et al. showed that CXCR3 played a critical role in resistance to sorafenib therapy by modifying the AMPK pathway, adipocytokine signaling and lipid peroxidation (Ren et al., 2020). In addition, sorafenib treatment was found to increase hypoxia and SDF1α/CXCR4 expression in HCC cells and animal tumor models, whereas inhibition of the SDF1α/CXCR4 pathway overcame the sorafenib resistance in HCC (Chen et al., 2014; Chen et al., 2015). Moreover, co-delivery of sorafenib and mifepristone using CXCR4-targeted PLGA-PEG nanoparticles vanishs sorafenib resistance in CXCR4-expressing HCC (Zheng et al., 2019). Recently, a newly discovered CXCR4 antagonist, BPRCX807, enhances the clinical efficacy of sorafenib (Song et al., 2021). Further studies are warranted to determine whether other chemokines are involved in sorafenib resistance.

## 5 Conclusions and perspectives

Although sorafenib resistance is an important clinical challenge for liver cancer treatment, the underlying mechanisms of sorafenib resistance are complex and still need to be explored. It has been reported that EMT, epigenetic regulation, cancer stem cells, transport processes, autophagy and the crosstalk between the PI3K/AKT and JAK-STAT pathways are involved in sorafenib resistance (Tovar et al., 2017; Tang J et al., 2020). Emerging evidence suggests that the TME plays an essential role in sorafenib resistance. It is worth noting that the therapeutic effect of sorafenib is significantly improved when combined with the drugs targeting hypoxic TME, tumor-associated immune suppressor cells or immunosuppressive factors (Table 2). However, the combination therapy is far from satisfactory since there are several reasons that may affect the poor clinical efficacy: 1) TME is a complex network participated by many elements. However, current studies tend to focus on a single cell type or factor and ignore the mutual regulation of the entire TME, especially the immunosuppressive microenvironment. Thus, the conclusions drawn from these studies are often incomplete or even contradictory. 2) The constructed drug-resistant cell lines and *in vivo* drug-resistant models often differ from the actual drug resistance observed in patients, which is also the most prominent problem in our study. Therefore, sorafenib resistance model should be improved or reestablished.

**TABLE 2:** Therapeutic approaches target TME to overcome sorafenib resistance in HCC.

Drugs	Targets	Cell lines/animal models/patients	References
miR-374b	PKM2-mediated glycolysis pathway	Hep3B-sorafenib resistance cells and HCCLM3-sorafenib resistance cells Hep3B-sorafenib resistant SCID mice subcutaneous HCC model	Zhang et al., (2019)
Simvastatin	HIF-1α/PPAR-γ/PKM2-mediated glycolysis	LM3-sorafenib resistance cells, LM3-sorafenib resistant nude mice subcutaneous HCC model	Feng J et al., (2020)
Proanthocyanidin B2	PKM2/HSP90/HIF-1α	LO2, HCC-LM3, SMMC-7721, Bel-7402, Huh-7 and HepG2 cells LM3 BALB/C nude mice subcutaneous HCC model	Feng et al., (2019)
Dauricine	PKM2, HK2	HepG2, Huh-7, Hep3B, Hepa1-6, H22andHL-7702 cells Huh-7 cells athymic nude mice subcutaneous HCC model	Li et al., (2018)
Genistein	GLUT1,HK2	HCC-LM3, SMMC-7721, Hep3B, Bel-7402, Huh-7 and LO2 cells HCC-LM3 cells BALB/C nu/nu mice subcutaneous HCC model	Li et al., (2017)
Aspirin	PFKFB3	HCC-LM3, SMMC-7721, Hep3B, Bel-7402, Huh7, QSG-7701 and LO2 cells	Li et al., (2017)
MK2206-2HCI	RIT1/PI3K/P38MAPK/AKT	CRL-8024 cells CRL-8024 (EV/RIT1 overexpression)cells BALB/C nude mice subcutaneous HCC model Huh7 (EV/RIT1 knockdown) cells BALB/C nude mice subcutaneous HCC model	Sun et al., (2022)
Verteporfin	YAP-IGF-1R signaling	HepG2215 and Hep3B cells/HepG2215-sorafenib resistance and Hep3B-sorafenib resistance cells HepG2215-sorafenib resistant NOD-SCID mice subcutaneous HCC models	Ngo et al., (2021)
Melatonin	mTORC1/p70S6K/HIF-1α	Hep3B cells	Prieto-Dominguez et al., (2017)
Rhizoma Paridis saponins	mRNA of HIF-1α	H22 cells Kunming mice subcutaneous HCC model	Yao et al., (2018)
2ME2	HIF-1α,HIF-2α	Huh-7 and HepG2 cells Huh-7 cells BALB/c nude mice subcutaneous HCC model	Ma et al., (2014)
Sodium orthovanadate	HIF-1α,HIF-2α	HepG2, Hep3B and SK-Hep-1 cells/HepG2-sorafenib resistance and Huh7-sorafenib resistance cells Huh7-sorafenib resistant BALB/c-nu/nu mice subcutaneous HCC model	Jiang et al., (2018)
Celecoxib and Meloxicam	COX-2/PGE2/COX-2	Huh-7, Hep3B, HepG2 and SMMC-7721 cells Huh-7 and Hep3B cells nude mice subcutaneous HCC model	Dong et al., (2019)
Metformin	HIF-2α	MHCC97H cells/MHCC97H cells orthotopic xenograft model	You et al., (2016)
MK2206-2HCI	RIT1/PI3K/P38MAPK/AKT	CRL-8024 cells CRL-8024 (EV/RIT1 overexpression)cells BALB/C nude mice subcutaneous HCC model Huh7 (EV/RIT1 knockdown) cells BALB/C nude mice subcutaneous HCC model	Sun et al., (2022)
Verteporfin	YAP-IGF-1R signaling	HepG2215 and Hep3B cells/HepG2215-sorafenib resistance and Hep3B-sorafenib resistance cells HepG2215-sorafenib resistant NOD-SCID mice subcutaneous HCC models	Ngo et al., (2021)
Melatonin	mTORC1/p70S6K/HIF-1α	Hep3B cells	Prieto-Dominguez et al., (2017)
Rhizoma Paridis saponins	mRNA of HIF-1α	H22 cells Kunming mice subcutaneous HCC model	Yao et al., (2018)
IRD-αCD206	TAMs	4T1cells, 4T1 cells female BALB/c mice subcutaneous breast cancer model	Zhang C et al., (2016)
IFN-α	Shifting the M2-like polarization of TAM	Hepa1-6 HCC and Huh7 HCC cells Hepa1-6 cells C57BL/6 mice subcutaneous HCC model	Zhang et al., (2021)
Compound kushen injection	Polarization TAMs to M1	Hepa1-6 tumor cells orthotopic HCC model Hepa1-6 and LPC-H12 cells nude mice subcutaneous HCC models	Yang et al., (2020a)
NRF-2/MicroRNA-1	NRF-2/miR-1/PD-L1	Hep3B and HepG2 cells/Hep3B sorafenib resistance cells and HepG2 sorafenib resistance cells Hep3B sorafenib resistant and HepG2 sorafenib resistant BALB/C nude mice subcutaneous HCC model	Li D et al., (2020)
Avelumab	PD-L1	Advanced HCC patients	Lee et al., (2021)
SB431542	TGF-β1-Mediated EMT	Hep3B and PLC/PRF/5 cells	Shrestha et al., (2021b)
Valproic acid	TGF-β/ERK/AKT	HepG2 and PLC/PRF/5 cells	Matsuda et al., (2014)
MiR-101	DUSP1/TGF-β	HepG2 and Huh7 cells	Wei et al., (2015)
Ulinastatin	TNF-α/NF-κB/EMT	HepG2, SK-HEP-1, and Huh-7 Hep3B and PLC/PRF/5 HCC cells SK-HEP-1 cells BALB/c athymic nude mice subcutaneous HCC model	Tan et al., (2019)
S3I-201	STAT3	Hepa1-6, Huh7 and Hep3B cells Orthotopic HCC mouse model with chronic liver injury	Jiang et al., (2021)
Nanaomycin A	IL-6/STAT3/DNMT3b/OCT4/DNMT1	Hep3B, HepG2 and Huh7 cells/Hep3B and HepG2.2.15 sorafenib resistance cells	Lai S.C et al., (2019)
Celecoxib	JAK2/IL-6/STAT3	Hep3B, HepG2, Huh-7, SNU-387 and SNU-449 cells	Liu et al., (2011)
747	CCL2/CCR2	Hepa1-6, THP-1, HepG2, LPC-H12, 7702, BEL-7404, SMMC-7721 and PVTT-1 cells Hepa1-6 and LPC-H12 cells BALB/c athymic nude mice subcutaneous HCC models	Yao et al., (2017)
C-021	CCR4	Orthotopic HCC mode	Gao et al., (2022)
C-021	TNF-α-RIP1-NF-κB/CCL22	Hepa1–6 cells, MHCC97 L MHCC97H, HepG2 and HepG2.2.15 cells Hepa1–6 cells BALB/c nude mice subcutaneous HCC models	Gao et al., (2020)
Metapristone	SDF-1/CXCR4 axis	HepG2, Huh7, and SMMC-7721 cells SMCC-7721 cells BALB/c nude mice subcutaneous HCC models	Zheng et al., (2019)
BPRCX807	CXCL12/CXCR4	HCA-1 and JHH-7 cells/orthotopic HCA-1 model JHH-7 cells nude mice subcutaneous HCC models DEN/CCl4-induced liver fibrosis associated HCC model	Song et al., (2021)

Since sorafenib is still the primary treatment for advanced HCC, it is of great importance to continue investigating the potential mechanisms of sorafenib resistance in HCC treatment. We believe that the combination therapy using sorafenib and TME-targeting drugs will be an effective strategy to overcome sorafenib resistance and improve outcome in patients with HCC.
